# The incidence of myopericarditis in patients with COVID-19

**DOI:** 10.34172/jcvtr.2021.36

**Published:** 2021-08-11

**Authors:** Atoosa Mostafavi, Seyed Abdol Hussein Tabatabaei, Somayeh Zamani Fard, Fatemeh Majidi, Abbas Mohagheghi, Shahin Shirani

**Affiliations:** Department of Cardiology, Tehran University of Medical Science, Dr Ali Shariati Hospital, Tehran, Iran

**Keywords:** Pericarditis, Pericardial Effusion, Myocarditis, Myopericarditis, COVID-19, SARS-COV-2

## Abstract

***Introduction:*** SARS-COV-2 can affect different organ systems, including the cardiovascular system with wide spectrum of clinical presentations including the thrombotic complications, acute cardiovascular injury and myopericarditis. There is limited study regarding COVID-19 and myopericarditis. The aim of this study was to evaluate myopericarditis in patients with definite diagnosis of COVID-19.

***Methods:*** In this observational study we analyzed the admitted patients with definite diagnosis of COVID-19 based on positive RT-PCR test. Laboratory data, and ECG changes on days 1-3-5 were analyzed for sign of pericarditis and also QT interval prolongation. Echocardiography was performed on days 2-4 and repeated as necessary, and one month after discharge for possible late presentation of symptom. Any patient with pleuritic chest pain, and pericardial effusion and some rise in cardiac troponin were considered as myopericarditis.

***Results:*** A total of 404 patients (18-90 years old, median = 63, 273 males and 131 females) with definite diagnosis of COVID-19 were enrolled in the study. Five patients developed in-hospital pleuritic chest pain with mild left ventricular dysfunction and mild pericardial effusion and diagnosed as myopericarditis, none of them proceed to cardiac tamponade. We found no case of late myopericarditis.

***Conclusion:*** Myopericarditis, pericardial effusion and cardiac tamponade are rare complication of COVID-19 with prevalence about 1.2 %, but should be considered as a possible cause of hemodynamic deterioration.

## Introduction

Corona virus disease 2019(COVID-19) an emerging disease which has grown to a worldwide pandemic affect the different organ systems, primarily infects the lung. Not all manifestation of disease have been fully understood.Most infected people will develop mild disease and recover spontaneously. Evidence shows the virus can affects the other organs, including the cardiovascular system with wide spectrum of clinical presentation as the acute cardiovascular injury, myocarditis, thromboembolic presentation including cerebrovascular accident, acute coronary syndrome, pulmonary thromboembolism and also small reports of pericardial effusion and clinical tamponade.^1,2,3,4^ The mechanisms responsible for cardiovascular damage have not been elucidated and some possibilities exist for such vulnerability. Increased myocardial oxygen demand in the setting of hypoxemia and acute inflammation are concerned the main mechanisms for acute coronary syndrome.^5^

Level of Troponin can be used to screen for potential myocardial damage, however not all of the patients with raised troponin suffered of acute coronary syndrome. Other possible mechanism is right ventricular (RV) strain due to significant lung damage or pulmonary thromboembolism, direct myocardial injury and inflammation resulting in myocarditis or myopericarditis. Viral infections are known as the main cause of pericarditis. Since evidence shows that Corona Virus has a tropism for myocardium and pericardium and virus has been isolated in pericardial fluid,^6^ by theory we might encounter a high number of patients with myopericarditis. None of the published data have been evaluated the incidence of myopericarditis, pericarditis and pericardial effusion in patients affected by SARS-COV-2 as the dominant complication, and in this study, we sought to assess the incidence of theses complications.

## Materials and Methods

In the presented observational study a total of 404 patients who were admitted to ICU and non-ICU COVID department of Dr Shariati Hospitals, Tehran, Iran and had positive RT-PCR nasopharynx swab test and had some lung involvement were enrolled. The index date was 1 August 2020 (Start of study) until 1 November 2020.The individuals were eligible if they were aged 18 years or older with positive RT-PCR test. Exclusion criteria were prior history of pericardial disease, poor echocardiographic image quality, active rheumatological disorders, end stage renal disease and /or glomerular filtration rate (GFR) less than 30 mL/min/1.73m, active cancer and uncontrolled hypothyroidism. The patients were enrolled into the study through consensus method.

Blood sampling was done on the first day to check the following: Complete blood count with differential (CBC with diff), Erythrocyte sedimentation rate (ESR), C-reactive protein (CRP) and Troponin I (TnI).

ECG was taken and analyzed on the days 1, 3, 5 and as necessary and analyzed for any ST_T changes, rhythm abnormality and QT interval duration. Transthoracic echocardiography was performed at days 2-4 and repeated as needed in the case of pleuritic chest pain or hemodynamic compromise, using 5-1 transducer of Sono Site -M-Turbo portable ultrasound system. All alive patients were followed up to one month after discharge by phone call for possible late presentation of symptom and all patients with LV systolic dysfunction and rise in cardiac troponin level, underwent late coronary angiogram or CT- angiography for possible coexistence of coronary artery disease.

Left atrial anteroposterior and left ventricular(LV) diastolic and systolic dimensions were measured from parasternal long axis view and right ventricular(RV) and right atrial (RA)sizes were measured from 4 chamber view accordance to American and European guideline 2015. ^7^ LV systolic function was assessed via Simpson method. Pulmonary artery pressure was measured by tricuspid (TV) peak systolic velocity adding to RA pressure. The presence or absence of pericardial effusion was examined from different views. Any patient with pleuritic chest pain, and pericardial effusion and some rise in cardiac troponin were considered as myopericarditis.

The Study was approved by the Ethics committee of Research Department of Tehran university of Medical Science. Categorical variables were expressed as proportion (%) and continuous data as mean ± SD or median as appropriate. For comparing mean in distributed continuous variable, unpaired T-test or the one-way analysis of variance (ANOVA) or χ^2^ tests were used. A P-value of less than 0.05 was considered significant. The statistical analyses were performed using the SPSS software, version 22.0 (SPSS, Inc, Chicago, IL).

## Results

The prospective study was conducted on 404 patients (273 males and 131 females) with definite diagnosis of COVID-19 based on positive RT-PCR test and variable degree of lung involvement. Patients aged 18-90 years old (mean total age = 58.36 y, mean male age = 59.72 y, mean female age = 57.00 y, median = 63).

160 patients (39.6%) had past history of hypertension (HTN) and 120 patients (29.7%) had previous history of diabetes mellitus.

Four patients had mitral valve prosthesis, 30 patients had history of Coronary Artery Bypass Grafting (CABGs), and 18 had chronic obstructive lung disease, 24 cases with chronic kidney disease, 4 with history of kidney transplantation and 10 patients with history of malignancy.

Baseline characteristics are summarized in Table 1.

**Table 1:** Demographic data of studied population

	**Number(n)**	***P*** ** value**
Age(years) (n = 404)	< 50 :n = 123	0.000*
	50-70:n = 196	
	> 70:n = 85	
Gender	Male: n = 273	0.000*
	Female: n = 131	
**Past medical history**	**Number of cases**	**Percent (%)**
DM	120	29.7
HTN	160	39.6
DLP	35	8.6
PCI	30	7.4
CABG	30	7.4
MVR	4	0.9
Malignancy	10	2.4
COPD	18	4.4
CKD	24	5.9
KT	4	0.9

Abbreviations: CABG, coronary artery bypass grafting; COPD, chronic obstructive lung diseas; CKD, Chronic kidney diease; DM, diabetes Mellitus, dyslipidemia; HTN, hypertension; KT, kidney transplantation; MVR, mitral valve replacement; PCI, percutaneous coronary intervention * *P* less than 0.05

The most presentation symptoms were dyspnea (73.4%) and cough (69.2 %).Other complaints as headache, diarrhea, myalgia, abdominal pain were less frequent. The first presentation of 13 patients was typical, anginal chest pain and were diagnosed as ST elevation myocardial infarction and referred for primary PCI. About 2 % of all COVID-19 patients were totally asymptomatic and were discovered incidentally. Mean duration of admission was 7 ± 2 days.

Patients had variable clinical course. Five out of 404 patients had in-hospital pleuritic chest pain with laboratory and echocardiographic features of myopericarditis such as mild LV dysfunction (40 < LVEF < 55%) and/or some rise in cardiac troponin I and mild pericardial effusion, without angiographically significant stenosis, although they had no ECG features in favor of pericarditis. D-dimer level was checked for all patients with pleuritic chest pain for possible diagnosis of pulmonary embolism, none had positive result. We found no case of late myopericarditis.

Patients with myopericarditis tended to be female (4/1), and hypertension was the most prevalent cardiovascular risk factor (HTN: 3, DM: 2, DLP and smoking: Zero cases).

Laboratory, ECG and echocardiographic data in patients with myopericarditis compared to non-myopericarditis patients are summarized in Table 2.

**Table 2:** Laboratory, ECG and echocardiographic data in patients with myopericarditis compared with non-myopericarditis patients

	**MP** **n (%)**	**non-MP** **n(%)**	***P*** ** value**
WBC count > 10 x 10^9^/l	1(20)	73(18.2)	0.979
Neutrophil (> 70 %)	1(20)	69(17.2)	0.224
ESR> 25 mm/h	1(20)	81(20.3)	0.824
CRP> 10 mg/l	2(40)	169(42.3)	0.493
TnI > 1ng/mL	5(100)	19(4.7)	0.000^*^
Heart rate (beat/min) < 60 60-100 > 100	0(0) 1(20) 4(80)	99(24.8) 290(72.6) 15(3.7)	0.016^*^
ST-T change: ST depression ST elevation T inversion	2(40) 0(0) 1(20)	104(26.0) 13(3.2) 95(23.8)	0.000^*^
QT interval prolongation(msec)	4(80)	23(5.7)	0.000^*^
LV dysfunction	5(100)	32(8.0)	0.034^*^
RV dysfunction	0(0)	34(8.5)	0.479
Pericardial effusion	5(100)	4(1.0)	0.000^*^

Abbreviations: CRP, C-reactive protein; ESR, erythrocyte sedimentation rate; MP, myopericarditis; non-MP, non myopericarditis; LV, left ventricle; RV, right ventricle; TnI, troponin I * *P* value less than 0.05

Clinical presentation, ECG and echocardiography and follow up of 5 cases with diagnosis of myopericarditis are summarized in Table 3.

**Table 3:** Clinical presentations, ECG, echocardiography and follow up of 5 cases with diagnosis of myopericarditis

**Case** **(n)**	**Gender** **(M/F)**	**age** **(y)**	**PMH**	**symptom 1**	**ECG 1**	**Echo1**	**Symptom 2**	**ECG2**	**Echo2**	**TnI** **(P/N)**	**Out come**
1	F	39	HTN	Dyspnea/fever	Normal	EF = 62%/No PE	Pleuritic CP	Sinus tachycardia, Long QT	EF = 46% Minimal PE	P	ICU ad./Discharge
2	F	43	DM	Cough/Dyspnea/fever	Sinus tachycardia	EF = 58%/No PE	Pleuritic CP	Sinus tachycardia,	EF = 46% Minimal PE	P	Discharge
3	F	52	DM,HTN	Cough/Dyspnea	Normal	EF = 53%/No PE	Pleuritic CP	Sinus tachycardia, Inverted T, Long QT	EF = 50%/Minimal PE	P	ICU ad/Death
4	F	41	HTN	Dyspnea/fever	Normal	EF = 61%/No PE	Aggregation of dyspnea/Pleuritic CP	ST depression, Long QT	EF = 48%/Mild PE	P	Discharge
5	M	29	Down syndrome	Cough/Diarrhea	Sinus tachycardia	EF = 68%/No PE	Low BP/Chest pain	Sinus tachycardia, ST depression, Long QT	EF = 49%/mild PE	P	ICU ad/Discharge

Abbreviations : CP, chest pain; DM, diabetes mellitus; EF, Ejection fraction; ICU ad, ICU admission; HTN, hypertension; M/F, male/female; P/N, positive/negative; PE, pericardial effusion

All 5 patients with diagnosis of myopericarditis had high level of cardiac TnI, and mild pericardial effusion, none of them had massive pericardial effusion and tamponade. ESR and CRP levels and total WBC count and differentiation were not significantly different with other patients.

Patients with myopericarditis had higher heart rate and longer corrected QT with no arrythmia except for mild sinus tachycardia. No specific ST-T changes were observed in patients with myopericarditis and no patient had typical ST elevation and PR depression. None of patients developed to cardiac tamponade and existence of pericardial effusion had no impact on mortality. Only one out of 5 patients ended to death due to progression of lung involvement and decline of O2 saturation.

## Discussion

COVID-19, an infectious disease caused by newly discovered Corona virus, affecting more than 111 millions people around the world up to 20 February 2020. The findings of several case reports indicate that the patient of all ages with current or previous infection can develop a hyperinflammatory syndrome and SARS-COV-2 has been found in multi organs including the heart, liver, brain, kidney and gastrointestinal tract.^8^ A growing number of evidence shows many people with COVID-19 survivors will experience short and long term heart damage, even without underlying heart disease and even with mild disease.

The cardiovascular complication reported are acute cardiac injury, arrythmia and cardiac insufficiency.^9^ The exact mechanism for cardiovascular problem are still unknown but some proposed mechanism are as follows; Direct viral involvement of cardiomyocyte, systemic inflammation, mismatch of demand and supply, sepsis leading to disseminated intravascular coagulation (DIC), coronary artery plaque rupture and superimposed thrombosis, side effect of medication and electrolyte imbalance.^10^

SARS-COV-2 stimulates the immune system which is responsible for viral elimination and recovery. Evidence suppose the hypothesis that the clinical presentation of COVID-19 is as a result of variable immune responses to the virus.^11^ Blanco-Melo et al showed that SARS-COV-2 induces a particular signature and leads to significant induction of multiple proinflammatory chemokines, IL-1B,IL6,TNFand IL1RA and the serum levels of theses cytokines which are elevated.^12^

Myocarditis is an inflammatory process secondary to inflammatory infiltration and myocardial damage.IL6 is a mediator of cytokine storm that cause T- lymphocyte activation and T- cell mediated cytotoxicity. ^13,14^ Pericardium also can be involved in inflammatory reactions and results in pericardial fluid accumulation. Karatolios and colleagues assessed pericardial and serum level of VEGF, bFGF, IL-1*β* and TNF-*α* by ELIZA in patients with viral and auto reactive pericardial effusion and concluded that the level of VEGF and bFGF are elevated in patients with inflammatory origin of PE.^15^ Theoretically, by considering the inflammatory process of SARS-COV-2, we expect the incidence of myopericarditis to be raised in COVID-19. O’ Gallagher et al reported the approximate 10% incidence of pericardial effusion in COVID-19 associated myocarditis, a complication that said to be uncommon.^16^ Based on the King‘s College Hospital(London UK) experience, patients with previous cardiac diseases are at increased risk of developing myopericarditis. However we didn’t find this association and the total incidence of pericardial effusion in our study population was about 1.2 % and all 5 patients had no history of cardiovascular disease.

It is important to know that COVID-19 can involve any organ without any pulmonary related sign or symptom. Amoozegar et al reported a 56 year-old-male who presented with complaint of recent onset dyspnea and chest pain without any associated fever and cough with large pericardial effusion but with no evidence of cardiac tamponade in echocardiography and ultimately diagnosed as COVID-19.^17^ There are some other reports of large pericardial effusion and cardiac tamponade as the presentation of COVID-19.^18,19^ In an international registry of patients with suspected and documented cases of COVID-19, 1216 cases were evaluated for any cardiac complications and only 1% of the cases had cardiac tamponade.^20^ In our study only 5 patients had mild pericardial effusion and none of them developed to cardiac tamponade. Different clinical manifestation seems to be correlated to immuno- globulin profile of patients. In patients with moderate infection the profile indicate a protective T cell response, in contrast in patients with severe infection it shows an exacerbate of systemic inflammation and sign of T cell exhaustion.^11.^ Ristić AD et al compared the pericardial cytokines in neoplastic, autoreactive and viral pericarditis and found the cytokine pattern in various diseases is different. In that study increased TNF-alpha level and high TNF-alpha/ Low TGF- beta1 pattern was found in viral pericardial effusion rather than neoplastic and autoreactive ones.^21^ TNF-alpha is an important cytokine in nearly all acute inflammatory reaction is increased significantly in patients with COVID-19 but couldn’t distinguish moderate versus severe disease presentation but is correlated with end organ damage.^22^ Whether different pattern of cytokines expression is the cause of distinct clinical presentation, such as existenced of pericardial effusion should be confirmed in in future studies.

In general, pericardial effusion and cardiac tamponade are not a prevalent complication of COVID-19, it should be considered as a possible cause of hemodynamic deterioration and decline of blood pressure and it seems mandatory to do follow up echocardiography for probable late development of constrictive pericarditis.

## Conclusion

Myopericarditis, pericardial effusion and cardiac tamponade are rare complication of COVID-19 with prevalence about 1.2 %, but should be considered as a possible cause of hemodynamic deterioration, follow up echocardiography should be done for evaluation of possible late complications such as late cardiac tamponade and constrictive pericarditis. Whether different pattern of cytokines expression is the cause of distinct clinical presentation, such as existence of pericardial effusion should be confirmed in future studies.

## Acknowledgments

We hereby express our thanks to Sakineh Momennia for her assistance.

## Competing interests

None.

## Ethical approval

The study was approved by ethical committee of Tehran University of Medical Sciences (No. IR.TUMS.MEDICINE.REC.1400.253

## Funding

The author(s) received no financial support for the research, authorship, and/or publication of this article.
